# Tooth Loss Related with Prevalence of Metabolic Syndrome in a General Urban Japanese Population: The Suita Study

**DOI:** 10.3390/ijerph19116441

**Published:** 2022-05-25

**Authors:** Takahiro Ono, Satoshi Kato, Yoshihiro Kokubo, Yoko Hasegawa, Takayuki Kosaka, Yoshinobu Maeda, Tomonori Okamura, Yoshihiro Miyamoto, Kazunori Ikebe

**Affiliations:** 1 Division of Comprehensive Prosthodontics, Faculty of Dentistry & Graduate School of Medical and Dental Sciences, Niigata University, Niigata 951-8514, Japan; cem17150@dent.niigata-u.ac.jp; 2 Department of Prosthodontics, Gerodontology and Oral Rehabilitation, Osaka University Graduate School of Dentistry, Suita 565-0871, Japan; satoshi.katun@gmail.com (S.K.); kosaka.takayuki.dent@osaka-u.ac.jp (T.K.); ymaeda@dent.osaka-u.ac.jp (Y.M.); ikebe.kazunori.dent@osaka-u.ac.jp (K.I.); 3 Department of Preventive Cardiology, National Cerebral and Cardiovascular Center, Suita 564-8565, Japan; ykokubo@ncvc.go.jp; 4 Department of Preventive Medicine and Public Health, School of Medicine, Keio University, Tokyo 160-8582, Japan; okamura@z6.keio.jp; 5 Open Innovation Center, National Cerebral and Cardiovascular Center, Suita 564-8565, Japan; miyamoty@ncvc.go.jp

**Keywords:** tooth, oral health, metabolic syndrome, cardiovascular disease, cerebrovascular disease

## Abstract

We examined whether the number of teeth could be a surrogate marker for metabolic syndrome (MetS) risk in cross-section. A total of 3771 individuals from the general urban Japanese population (1690 men, 2081 women; mean age 67.1 ± 11.0 years) participated in this study. Participants were diagnosed with MetS with three or more components hypertension, hyperglycemia, lipid metabolism abnormality, and abnormal abdominal girth. Questionnaires were administered to determine the number of teeth, smoking status, drinking status, and past illnesses. To clarify the relationships between the number of teeth and the presence of MetS components, we divided subjects into two groups: those with less than 20 residual teeth and those with 20 or more, then statistical analyses (Mantel-Haenszel tests and logistic regression analysis) were performed. MetS were higher for those with ≤19 teeth than those with ≥20 teeth when examining all participants and women-only groups. Hyperglycemia, low HDL cholesterol, high triglycerides, and diagnosis with MetS were all significantly higher in the ≤19 teeth group for both sexes combined and for women. These results suggest that less than 20 teeth may be a surrogate marker for MetS risk, but further studies on gender differences and pathological background are needed.

## 1. Introduction

It has been reported that age-related tooth loss causes poor masticatory ability, which causes malnutrition and poses a risk of general frailty and leads to poor nutritional balance through changes in food choices [1,2,3]. Recently, Fushida et al. showed in a follow-up study that reduced objective masticatory performance poses a risk for the development of hypertension, triglyceride levels, hyperglycemia, and metabolic syndrome (MetS), a complex condition of overlapping hypertension, abdominal obesity, dyslipidemia, and hyperglycemia in the same individual [4]. This emphasizes the importance of masticatory performance assessment in health promotion to prevent cardiovascular disease. However, there is a drawback that objective and quantitative masticatory performance assessments can only be performed at specialized dental medical institutions.

Occlusal force and masticatory performance are known to depend on the number of teeth, and a decrease in the number of teeth has often been used as a surrogate marker for decreased masticatory function [5]. The Japanese Society of Gerodontology allows the use of fewer than 20 teeth (normally, the number of permanent teeth is 28 teeth, excluding wisdom teeth) in the diagnosis of decreased occlusal force [6], which is one of the sub-symptoms of oral hypofunction, in the absence of an occlusal force test device. Behind this, there is the “8020” movement aimed at achieving the goal of oral health, “keeping more than 20 teeth at the age of 80,” by the Japanese Ministry of Health, Labor and Welfare and the Japan Dental Association since 1990 [7]. In comparison between the 8020 achievers and the 8020 non-achievers, it has been reported that there was a significant difference in physiological indicators such as weight, heart rate, and survival rate [8]. Furthermore, several studies have reported that subjects with <20 teeth had a significantly higher mortality risk than those with ≥20 teeth [9,10].

Previous studies suggested that tooth loss affected nutritional intake by altering the food products consumed, consequently exacerbating the metabolic abnormalities [11,12]. Epidemiological studies have been carried out to investigate the relationships between the number of remaining teeth and heart disease [13,14,15], sclerosis of the aortic valve [16], and atherosclerosis [17]. These results have indicated a relationship between the number of missing teeth and the components of the metabolic syndrome (Mets). Some epidemiological studies have examined the relationship between the incidence of MetS and tooth loss [18,19]; however, as far as we know, there are no reports targeting middle-aged and older Japanese people. Therefore, in this study, we cross-sectionally examined whether less than 20 teeth are a surrogate marker for assessing the risk of MetS due to decreased masticatory function in a general urban Japanese population.

## 2. Materials and Methods

### 2.1. Research Subjects

The Suita study is a cohort study investigating a random sample from a general urban population that was initiated in 1999 to promote measures to prevent cardiovascular disease (CVD) in Japan. The study population includes 6485 people who participated in health check-ups at the National Cardiovascular Center from a primary cohort of 12,200 residents of Suita, a city in Japan, who were arbitrarily selected from the municipal population registry and divided into groups by sex and age in 1989, 1329 people who participated in health checks from among 3000 people selected in 1996 for a second cohort in the same way, and 546 members of a volunteer organization, for a total of 8360 people. As a general rule, participants in regular health check-ups every 2 years. The participants for the present study were 3771 people aged 40–96 years (1690 men, 2081 women; mean age 67.1 ± 11.0 years), who underwent a health check-up in 2005 and 2006. The study protocol was approved by the ethics committee of the National Cardiovascular Center (M25-032).

### 2.2. Questionnaire Survey

Standardized questionnaires were administered to subjects to determine the number of teeth, smoking status, drinking status, and past illnesses. For the number of teeth, subjects selected one of four options (0 teeth, 1–9 teeth, 10–19 teeth, or 20 or more teeth) for their current number of teeth. Subjects were divided into ≥20 teeth and ≤19 teeth groups. The rate of agreement between the examination by the dentist and the self-assessment of subjects was 92% for 711 subjects who participated in health check-ups for the Suita study. Hence, the same concordance rate was assumed for the self-reported number of teeth in the present study.

### 2.3. Survey on the Components of Metabolic Syndrome

Participants were instructed to fast from 9:00 p.m. the night before the check-up. The following day, a 4-mL blood sample was taken after 12 h of fasting; fasting blood sugar, high-density lipoprotein (HDL), and blood triglyceride levels were measured. Systolic blood pressure (SBP) and diastolic blood pressure (DBP) were measured twice in a seated position after at least 5 min of rest. Well-trained laboratory technicians measured abdominal girth in a standing position at the umbilical height to the nearest 1 cm.

### 2.4. Definition of Metabolic Syndrome

Diagnostic criteria for determining the presence of MetS were the criteria defined in uniform international standards based on the National Cholesterol Education Program/Adult Treatment Panel III [20]. It has been reported that Mets defined in this criteria are related to cardiovascular disease onset by the follow-up survey of the Suita study for 13 years [21]. Abnormal SBP of ≥130 mmHg and/or DBP of ≥85 mmHg indicated elevated blood pressure. Fasting blood glucose of ≥100 mg/dL indicated abnormal blood glucose levels. Triglycerides of ≥150 mg/dL and/or HDL cholesterol level of <40 mg/dL for men or <50 mg/dL for women indicated abnormal serum lipid levels. Since the Asian build differs from the Western build, abnormal abdominal girth was based on diagnostic criteria for Asia [22], ≥90 cm for men and ≥80 for women. Participants with three or more abnormal components from blood pressure, blood glucose, triglycerides, HDL cholesterol, and abdominal girth were diagnosed with MetS. Those who were taking medication for any of the MetS components at the time of the examination were diagnosed as abnormal even if the test values were within the normal range.

### 2.5. Data Analysis

Participants were divided into ≥20 teeth or ≤19 teeth groups. We divide the whole participant into two groups of men and women. Student’s *t*-test (parametric distribution) or Mann–Whitney U test (non-parametric distribution) were performed to compare age, abdominal girth, SBP, DBP, blood HDL cholesterol level, blood triglyceride level, and fasting blood glucose level between the groups. Chi-squared tests (Mantel-Haenszel tests) were performed to compare the rates of subjects with abnormal MetS components and the rate of MetS between the ≥20 teeth group and the ≤19 teeth group for both sexes together and for each sex separately.

Logistic regression analysis was performed to investigate the relationship between the number of teeth and MetS, MetS components (Waist, Blood pressure, HDL cholesterol, triglyceride, and fasting blood glucose level) or diagnosed with MetS as the dependent variable (Normal = 0/Abnormal = 1), the number of teeth (≥20 teeth/≤19 teeth:0/1) as the independent variable, and confounding variables of age, current drinker, current smoker, and past illness (hypertension, hyperglycemia, hyperlipidemia) as moderator variables (forced entry). The moderator variable of sex was added to the all-participants model. The statistical level of significance was set to 5% for all analyses carried out with SPSS16.0 J and Aimos 13.0.

## 3. Results

Table 1 shows the results of comparing age and Mets components between the ≥20 teeth and ≤19 teeth groups for all participants together and for each sex. Overall, the group with 19 or fewer teeth was significantly older than the group with 20 or more teeth. There was no significant difference between the ≥20 teeth and ≤19 teeth groups in fasting plasma glucose, triglyceride, and LDL cholesterol for men. There was a significant difference in all components for women and total participant groups between ≥20 teeth and ≤19 teeth groups. The ≤19 teeth group showed a tendency for the Mets component to show an abnormal value than the ≥20 teeth group.

Risks diagnosed as Mets were significantly higher for those with ≤19 teeth for Women and Total participants (Table 2). The prevalence of hyperglycemia, low HDL cholesterol, and high triglycerides was higher in the ≤19 teeth group than in the ≥20 teeth group for Total and for women only but showed no differences between groups for any components for men.

**Figure 1:**
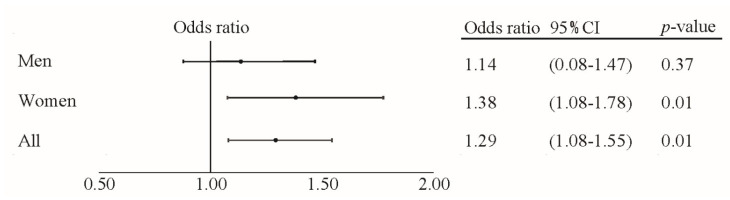
Forest plot shows the relationship between less than 20 teeth and MetS morbidity in men and women subjects.

Hyperglycemia, low HDL cholesterol, high triglycerides, and diagnosis with MetS were all significantly higher in the ≤19 teeth group for both sexes combined and for women by logistic regression analysis (Table 3 and Figure 1). The odds ratio (OR) was higher for women for each item. Among men, the OR tended to be higher for MetS and all abnormal components except obesity, but this tendency was not significant.

## 4. Discussion

The results of the present study demonstrate that there is an association between tooth loss and MetS, as well as MetS components (hyperglycemia, low HDL cholesterol, and high triglycerides) in the general urban Japanese 40 year and older population, suggesting that the number of teeth of 20 or less could be a surrogate marker in assessing the MetS risk due to decreased masticatory function. Decreased number of teeth could lead to decreased masticatory performance [5,23], and it might result in the group with decreased masticatory ability avoiding chewy foods and preferring soft foods [1,2,3,24], and resulting decrease in dietary fiber and Vitamins A and C intake and increase in the intake of carbohydrate [25]. These changes in nutritional intake affect the components of MetS, such as lipid and carbohydrate metabolism and blood pressure [26]. By linking these effects, a decrease in the number of teeth is considered to be a risk of developing MetS. However, when the results of this study are compared with the results of previous studies, some conflicting points are found, and future research topics are also found there.

In this study, there was a gender difference in the number of teeth and the risk of incidence of MetS and its components. When analyzing the results with men-only and women-only models showed that there were no significant associations between the number of teeth and MetS or abnormal MetS components among men, while having less than 20 teeth was significantly associated with higher rates of hyperglycemia, hyperlipidemia, low HDL cholesterol and MetS among women, similar to the results for both sexes combined. These results indicate that the association between tooth loss and MetS and lipid/carbohydrate abnormalities is particularly pronounced in women. However, Fushida et al. [4] followed 599 adults in their 50s and 70s for an average of 4.4 years in the same field as this study. They found that in men with a low objective masticatory performance at baseline, the risk of developing new MetS, hypertension, high blood glucose, and triglyceride levels was significantly higher, but no significant difference was observed in women.

It should be considered here that tooth loss is the starting point for masticatory deterioration as well as the endpoint of dental caries and periodontal disease. Multiple tooth loss due to periodontal disease can be interpreted as prolonged exposure to inflammatory cytokines. Cross-sectional studies have shown a link between MetS components (hypertension, diabetes, hyperlipidemia, etc.) [27], arteriosclerosis [28], hyperglycemia [29], hyperlipidemia [30], obesity [31]. It has also been reported that patients with periodontitis have a high prevalence of metabolic syndrome [16,32,33,34]. Regarding the relationship between periodontal disease and MetS, it was strong in men in a Korean study [35] and a Japanese longitudinal study [36], while it was strong in women in a US study [37] and another Japanese cross-sectional study [38]. Thus, there are conflicting reports on gender differences in the relationship between periodontal disease and MetS. In addition, several papers demonstrated that significant heterogeneity existed between men and women in developing the metabolic syndrome [39,40,41].

Therefore, regarding the relationship between the decrease in the number of teeth and MetS, in addition to the dental diseases that cause the decrease in the number of teeth, the influence of multiple factors such as the etiology of each component of MetS and the gender difference in the risk of developing lifestyle-related diseases need to be considered. And it is also considered that the interaction between these factors is also involved in the background of the conflicts seen in the research results so far. For example, postmenopausal hormone balance changes strongly affect women’s circulatory and metabolic mechanisms [42]. The number of teeth decreases dramatically in Japanese women during the menopausal period from their late 40s until their late 50s [42]. If severe periodontal disease and tooth loss during this period can be attributed to changes in nutritional balance caused by changes in eating habits, it is conceivable that the effects on women will be stronger than on men. On the other hand, women are more careful about their dietary habits than men [43], and in Japan, it has been reported that women cook more often than men [44]. These reports suggest that women are more likely to compensate for reduced masticatory performance through food selection and cooking ingenuity.

Hashimoto et al. [45], who conducted a follow-up study in the same field as this study, reported that decreased occlusal force increased the risk of new-onset of cardiovascular disease. Even if the number of teeth decreases, prosthodontic treatment can recover masticatory performance [46]. Even if the masticatory function deteriorates, it is possible to prevent the nutritional imbalance by devising cooking and appropriate nutritional guidance. Therefore, a reduced number of teeth can also be a meaningful marker because it suggests dental and nutritional approaches to prevent MetS and cardiovascular disease.

This study had several limitations. One of these was the sampling bias that the subject agreed to participate from a random sample of the general population in urban areas of Japan. Furthermore, because this is a cross-sectional study, it could not explain the causal relationship between tooth loss and the morbidity of MetS and its components. Although a longitudinal study has been conducted to elaborate the association between decreased masticatory performance and new morbidity of MetS, as mentioned above, a decreased number of teeth is not only the cause of decreased masticatory ability but also the result of periodontal disease. Therefore, it is also necessary to consider the “bidirectional possibility” that tooth loss is accelerated by systemic diseases associated with periodontal disease. In addition, there was a limitation that other oral health factors, brushing habits, denture wearing, saliva secretion, etc. was not taken into consideration. In the future, long-term studies that take these factors into consideration will make the significance of reducing the number of teeth as a health risk clearer.

## 5. Conclusions

In conclusion, less than 20 teeth may be a surrogate marker that associates the risk of developing MetS. Interpretation of its etiology requires long-term longitudinal studies based on both consideration of periodontal disease and decreased masticatory function. Although that should be taken into account, these results emphasize the need for medical-dental collaboration in the prevention of MetS and cardiovascular disease and provide health information that can be effectively utilized in the population approach.

## Figures and Tables

**Table 1:** Characteristics of subjects according to the number of teeth (≧20 teeth or ≦19 teeth).

	Men			Women			Total		
	≧20 Teeth	≦19 Teeth		≧20 Teeth	≦19 Teeth		≧20 Teeth	≦19 Teeth	
Characteristics	(*n* = 946)	(*n* = 744)		(*n* = 1336)	(*n* = 745)		(*n* = 2282)	(*n* = 1489)	
	Mean ± SD	Mean ± SD	*p* Value	Mean ± SD	Mean ± SD	*p* Value	Mean ± SD	Mean ± SD	*p* Value
	Median (IQR)	Median (IQR)		Median (IQR)	Median (IQR)		Median (IQR)	Median (IQR)	
Age	64.6 ± 10.3	72.9 ± 9.6	<0.001 *	62.5 ± 10.2	72.5 ± 9.1	<0.001 *	63.4 ± 10.3	72.7 ± 9.4	<0.001 *
(yrs)	65.0 (57.0–72.0)	74.0 (67.0–80.0)		63.0 (55.0–70.0)	73.0 (67.0–79.0)		64.0 (55.0–71.0)	74.0 (67.0–79.0)	
Waist	85.9 ± 8.0	84.9 ± 8.6	0.042 *	81.5 ± 9.1	83.3 ± 9.9	<0.001 *	83.3 ± 8.9	84.1 ± 9.3	<0.001 *
(cm)	86.0 (80.5–91.2)	85.4 (79.5–90.5)		80.8 (75.0–87.0)	83.3 (76.5–89.6)		83.1 (77.2–89.1)	84.5 (78.0–90.0)	
Diastolic blood pressure	78.4 ± 10.7	77.1 ± 10.7	0.012 *	72.5 ± 10.6	74.3 ± 10.8	<0.001 *	74.9 ± 11.0	75.7 ± 10.8	<0.040 *
(mmHg)	78.0 (71.0–85.6)	76.6 (69.5–84.5)		71.5 (64.5–79.5)	73.5 (67.0–81.5)		74.5 (67.0–82.5)	75.0 (68.2–82.7)	
Systolic blood pressure	124.5 ± 17.7	127.0 ± 18.7	0.003 *	118.2 ± 17.7	126.6 ± 19.9	<0.001 *	120.8 ± 17.9	126.8 ± 19.3	<0.001 *
(mmHg)	122.8 (112.0–135.1)	125.0 (113.0–139.0)		115.5 (105.0–129.5)	124.5 (111.5–139.5)		119.0 (107.5–132.5)	124.5 (112.5–139.0)	
HDL cholesterol	55.0 ± 14.4	53.3 ± 14.5	0.008 *	65.2 ± 14.7	61.5 ± 14.4	<0.001 *	61.0 ± 15.4	57.4 ± 15.0	<0.001 *
(mg/dL)	53.0 (46.0–62.0)	51.0 (43.0–61.0)		64.0 (55.0–74.0)	60.0 (51.0–70.0)		59.0 (50.0–70.0)	55.0 (46.0–67.0)	
LDL cholesterol	118.6 ± 28.2	116.8 ± 27.7	0.190	131.7 ± 29.5	127.4 ± 28.1	0.001 *	126.2 ± 29.7	122.1 ± 28.4	<0.001 *
(mg/dL)	119.4 (99.6–136.4)	116.6 (97.4–135.0)		131.8 (110.6–150.4)	124.8 (108.4–144.1)		125.4 (106.2–145.2)	121.0 (103.4–140.1)	
Fasting plasma glucose	105.0 ± 24.4	106.5 ± 23.2	0.069	96.2 ± 15.1	100.3 ± 18.4	<0.001 *	99.8 ± 20.0	103.4 ± 21.2	<0.001 *
(mg/dL)	99.0 (93.0–108.0)	100.0 (93.0–111.0)		94.0 (89.0–99.0)	96.0 (90.0–104.0)		95.0 (90.0–103.0)	98.0 (92.0–107.0)	
Triglyceride	116.0 ± 69.5	113.4 ± 67.9	0.595	92.8 ± 48.7	99.8 ± 47.9	0.002 *	102.5 ± 59.3	106.6 ± 59.2	0.001 *
(mg/dL)	97.0 (69.0–141.0)	96.0 (68.3–137.0)		82.0 (60.0–111.0)	89.0 (68.0–119.0)		87.5 (64.0–125.0)	92.0 (68.0–129.0)	

Normal distribution: Student *t*-test, abnormal distribution: Mann–Whitney U test. * *p* < 0.05.

**Table 2:** Association between the number of teeth (≧20 teeth or ≦19 teeth) and prevalence of abnormal MetS components and diagnosis with MetS.

Components of Metabolic Syndrome (MetS)		Men		Women		Total	
		≧20 teeth	≦19 Teeth	≧20 Teeth	≦19 Teeth	≧20 Teeth	≦19 Teeth
		(*n* = 946)	(*n* = 744)	(*n* = 1336)	(*n* = 745)	(*n* = 2282)	(*n* = 1489)
		No. of Participants (%)		No. of Participants (%)		No. of Participants (%)	
Metabolic Syndrome	Normal	748 (79.1)	587 (78.9)	1135 (85.0)	560 (75.2)	1883 (82.5)	1147 (77.0)
	Abnormal	198 (20.9)	157 (21.1)	201 (15.0)	185 (24.8)	399 (17.5)	342 (23.0)
	Odds ratio (CI)	1	1.14 (0.88–1.47)	1	1.38 (1.08–1.78)	1	1.29 (1.08–1.55)
	*p* value	-	0.33	-	0.01 *	-	0.01 *
Blood pressure (mmHg)	Systolic < 130 and Diastolic < 85	560 (59.2)	399 (53.6)	970 (72.6)	439 (58.9)	1530 (67.0)	838 (56.3)
	Systolic ≧ 130 or Diastolic ≧ 85	386 (40.8)	345 (46.4)	366 (27.4)	306 (41.1)	752 (33.0)	651 (43.7)
	Odds ratio (CI)	1	1.04 (0.84–1.29)	1	1.13 (0.92–1.40)	1	1.12 (0.97–1.30)
	*p* value	-	0.71	-	0.27	-	0.15
Fasting plasma glucose (mg/dL)	<100	502 (53.1)	365 (49.1)	1008 (75.4)	472 (63.4)	1510 (66.2)	837 (56.2)
	≧100	444 (46.9)	379 (50.9)	328 (24.6)	273 (36.6)	772 (33.8)	652 (43.8)
	Odds ratio (CI)	1	1.21 (0.98–1.50)	1	1.33 (1.07–1.65)	1	1.32 (1.14–1.53)
	*p* value	-	0.08	-	0.01 *	-	<0.01 *
HDL cholesterol (mg/dL)	Men ≧ 40 Women ≧ 50	845 (89.3)	637 (85.6)	1161 (86.9)	599 (80.4)	2006 (87.9)	1236 (83.0)
	Men < 40 Women < 50	101 (10.7)	107 (14.4)	175 (13.1)	146 (19.6)	276 (12.1)	253 (17.0)
	Odds ratio (CI)	1	1.37 (1.00–0.64)	1	1.41 (1.08–1.85)	1	1.37 (1.12–1.69)
	*p* value	-	0.05 *	-	0.01 *	-	<0.01 *
Triglyceride (mg/dL)	<150	742 (78.4)	601 (80.8)	1202 (90.0)	645 (86.6)	1944 (85.2)	1246 (83.7)
	≧150	204 (21.6)	143 (19.2)	134 (10.0)	100 (13.4)	338 (14.8)	243 (16.3)
	Odds ratio (CI)	1	1.05 (0.81–1.36)	1	1.48 (1.08–2.01)	1	1.28 (1.05–1.56)
	*p* value	-	0.72	-	0.02 *	-	0.02 *
Waist (cm)	Men < 90 Women < 80	654 (69.1)	539 (72.4)	613 (45.9)	275 (36.9)	1267 (55.5)	814 (54.7)
	Men ≧ 90 Women ≧ 80	292 (30.9)	205 (27.6)	723 (54.1)	470 (63.1)	1015 (44.5)	675 (45.3)
	Odds ratio (CI)	1	0.90 (0.72–1.13)	1	1.12 (0.91–1.38)	1	0.97 (0.84–1.12)
	*p* value	-	0.4	-	0.31	-	0.68

Mantel-Haenszel test adjusted by sex and age for total participants and adjusted by age for men and women. * *p* < 0.05.

**Table 3:** Adjusted association between the number of teeth (≧20 teeth or ≦19 teeth) and prevalence of abnormal MetS components and MetS.

Components of Metabolic Syndrome (MetS)		Men		Women		Total	
		Adjusted Odds Ratio (95% CI)	*p* Value	Adjusted Odds Ratio (95% CI)	*p* Value	Adjusted Odds Ratio (95% CI)	*p* Value
Blood pressure (mmHg)	≧20 teeth	1		1		1	
	≦19 teeth	1.11 (0.90–1.38)	0.33	1.15 (0.92–1.43)	0.23	1.15(0.98–1.34)	0.08
Fasting plasma glucose (mg/dL)	≧20 teeth	1		1		1	
	≦19 teeth	1.22 (0.98–1.51)	0.08	1.37 (1.09–1.70)	0.01 *	1.32 (1.13–1.53)	<0.01 *
HDL cholesterol (mg/dL)	≧20 teeth	1		1		1	
	≦19 teeth	1.23 (0.89–1.70)	0.21	1.40 (1.06–1.83)	0.02 *	1.32 (1.07–1.63)	0.01 *
Triglyceride (mg/dL)	≧20 teeth	1		1		1	
	≦19 teeth	1.06 (0.82–1.39)	0.65	1.47 (1.07–2.02)	0.02 *	1.25 (1.02–1.52)	0.03 *
Waist (cm)	≧20 teeth	1		1		1	
	≦19 teeth	0.91 (0.72–1.15)	0.42	1.12 (0.91–1.37)	0.31	0.99 (0.85–1.15)	0.89

Logistic regression analysis adjusted for age, current smoking, and current drinking and with/without medical history. * *p* < 0.05.

## Data Availability

The datasets presented in this article are not readily available because request for date disclosure will be granted at the discretion of the Facility Ethics Committee. Requests to access the datasets should be directed to Takahiro Ono, ono@dent.niigata-u.ac.jp.
